# Stereoselective α-fluoroamide and α-fluoro-γ-lactone synthesis by an asymmetric zwitterionic aza-Claisen rearrangement

**DOI:** 10.1186/1860-5397-1-13

**Published:** 2005-10-17

**Authors:** Kenny Tenza, Julian S Northen, David O'Hagan, Alexandra M Z Slawin

**Affiliations:** 1 School of Chemistry, University of St Andrews, North Haugh, St Andrews, Fife, UK KY16 9ST; 2 Onyx Scientific Ltd., Units 97/98 Silverbriar, Sunderland Enterprise Park East, Sunderland UK, SR5 2TQ

## Abstract

**Background:**

Asymmetric introduction of fluorine α-to a carbonyl has become popular recently, largely because the direct fluorination of enolates by asymmetric electrophilic fluorinating reagents has improved, and as a result such compounds are becoming attractive synthons. We have sought an alternative but straightforward asymmetric method to this class of compounds, utilising the zwitterionic aza-Claisen rearrangement by reacting α-fluoroacid chlorides and homochiral N-allylpyrrolidines as starting materials.

**Results:**

Treatment of N-allylmorpholine with 2-fluoropropionyl chloride under Yb(OTf)_3_ catalysis generated the zwitterionic aza-Claisen rearrangement product in good yield and demonstrated the chemical feasibility of the approach. For the asymmetric reaction, N-allyl-(*S*)-2-(methoxymethyl)pyrrolidine was treated with either 2-fluoropropionyl chloride or 2-fluorophenylacetic acid chloride under similar conditions and resulted in N-(α-fluoro-γ-vinylamide)pyrrolidine products as homochiral materials in 99% de. These products were readily converted to their corresponding α-fluoro-γ-lactones by iodolactonisation and in good diastereoselectivity.

**Conclusion:**

Molecules which have fluorine at a stereogeneic centre are finding increasing utility in pharmaceutical, fine chemicals and materials research. The zwitterionic aza-Claisen rearrangement proved to be an effective and competitive complement to asymmetric electrophilic fluorination strategies and provides access to versatile synthetic intermediates with fluorine at the stereogenic centre.

## Introduction

**Scheme 1:**
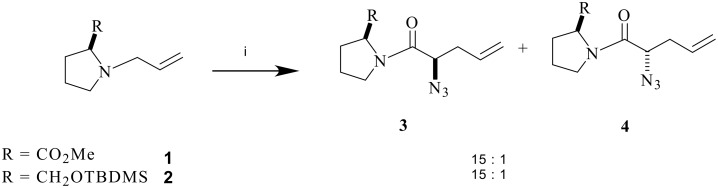
*Reagents*: i N_3_CH_2_C(O)F, AlMe_3_

The development of methods for the stereoselective introduction of the C-F bond, α-to a carbonyl group has been a significant and recent focus in organo-fluorine chemistry.[1–2] Most effort has involved enolate reactions with electrophilic fluorinating reagents, either using asymmetric enolates, [3–4] asymmetric fluorinating reagents[5–6] or asymmetric Lewis acids.[7–9] Most recently organocatalysis mediated asymmetric fluorinations have been explored[10] and this has resulted in the efficient preparation of α-fluoroaldehydes in high enantiomeric purity.[11] Successes in this area has advanced methodology in organofluorine chemistry considerably over the last decade or so.[1–2] In this paper we explore an alternative approach for the preparation of α-fluorocarbonyls using an asymmetric zwitterionic aza-Claisen rearrangement on appropriate fluorinated substrates, to generate α-fluoro-γ-vinyl amides and then α-fluoro-γ-lactones as the end products after iodolactonisation. In 1998 Nubbemeyer[12–13] reported on such aza-Claisen rearrangements using the N-allylproline ester 1 and the N-allylpyrrolidine ether 2 with the acid fluoride of azidoacetic acid to generate the α-azido-γ-vinyl amide diastereoisomers 3 and 4, with good diastereo control (~88%de) (Scheme 1).

With this background, it was envisaged that the aza-Claisen approach could be exploited to generate α-fluoro-γ-vinyl amide products from appropriate α-fluoroacid chlorides and suitable amines, to offer an alternative strategy to α-fluorocarbonyl compounds. Such products can be converted to γ-lactones by straightforward iodolactonisation.[14] γ-Lactones are a ubiquitious motif found in many natural product sand they are also useful templates for the synthesis of a wide range of bio-actives of pharmaceutical interest.[15] It is well known too that selective fluorination can improve pharmacokinetics and the fluorine substituent can often modify bio-activity in an advantageous manner.[16] For example in the structural series relevant to this study the α-fluorinated-γ-lactone **5** is a key intermediate in the synthesis of the *anti*-HIV nucleoside β-FddA^1^ **6**. [17–18]

## Results and discussion

**Scheme 2:**
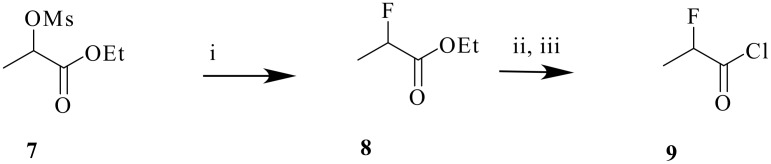
*Reagents*: i KF, DMF, 73%; ii NaOH, EtOH then aqHCl, 44%; iii (CO)_2_Cl_2_, 90%.

In order to undertake the appropriate zwitterionic aza-Claisen rearrangement reactions an efficient method for the production of the α-fluoro acid chloride substrates was required. A number of routes to 2-fluoropropionyl chloride **9** were explored but the method of choice involved nucleophilic fluorination of the mesylate **7** with KF to give ethyl 2-fluoropropionate **8** (Scheme 2).[19] Saponification and then treatment with phthaloyl dichloride gave **9** after distillation. 2-Fluorophenylacetyl chloride was prepared from phenylglycine as previously described.[20]

In the first instance a Yb(OTf)_3_ mediated aza-Claisen rearrangement using allyl morpholine **10** and acid chloride **9** was explored following MacMillan's protocol.[21] This proceeded smoothly to give the α-fluoroamide **11** in good yield although reduction of the equivalence of the Lewis acid below 0.5 resulted in poor conversions.

**Scheme 3:**
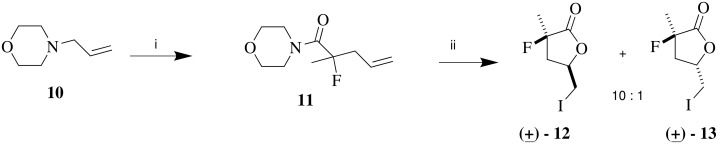
*Reagents*: i *i*Pr_2_EtN, Yb(OTf)_3_, **9**, DCM, 92%; ii I_2_, THF/ H_2_O, Na_2_S_2_O_3_, 82%.

**Scheme 4:**
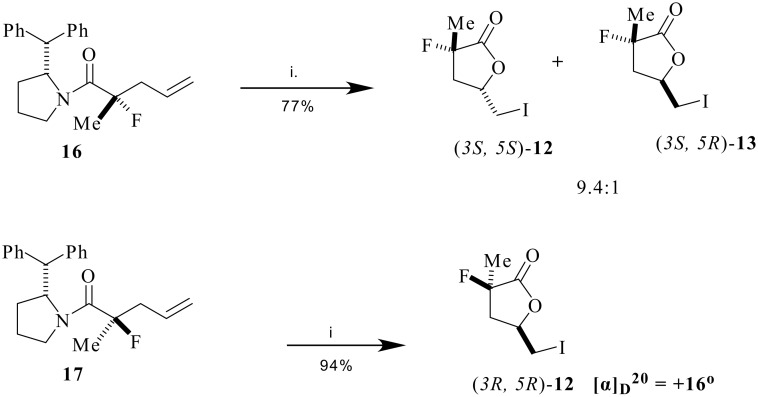
*Reagents* i. I_2_, THF/H_2_O.

Iodolactonisation of amide **11** afforded the α-fluoro-iodolactone **12** as the major diastereoisomer[12] in a mixture of **12** and **13** (10:1). Isomer **12** was assigned the *anti* stereochemistry by ^1^H-NMR nOe analysis as shown in Scheme 3, a conclusion which is entirely consistent with the literature.[22] An asymmetric variant of the reaction was then explored. In the first instance (*R*)-2-(diphenylmethyl)pyrrolidine **14**[23] was converted to allylamine **15** as a potential substrate for the aza-Claisen reaction. Subsequent treatment of allylamine **15** with 2-fluoropropionyl chloride, Hünigs base and Yb(OTf)_3_, generated the diastereoisomers **16** and **17** in a 3:1 ratio. The diastereoselectivity was not high and it could not be improved, even with more than 1 equivalent of the Lewis acid. Never-the-less, the diastereoisomers could be easily separated by chromatography to generate **16** and **17** as homochiral materials. The major diastereoisomer **16**, was then subjected to iodolactonization and this resulted in a stereoisomer mixture of (*3S, 5S*)-**12** and (*3S, 5R*)-**13** in a ratio of 9.4:1 (Scheme 4).

**Scheme 5:**
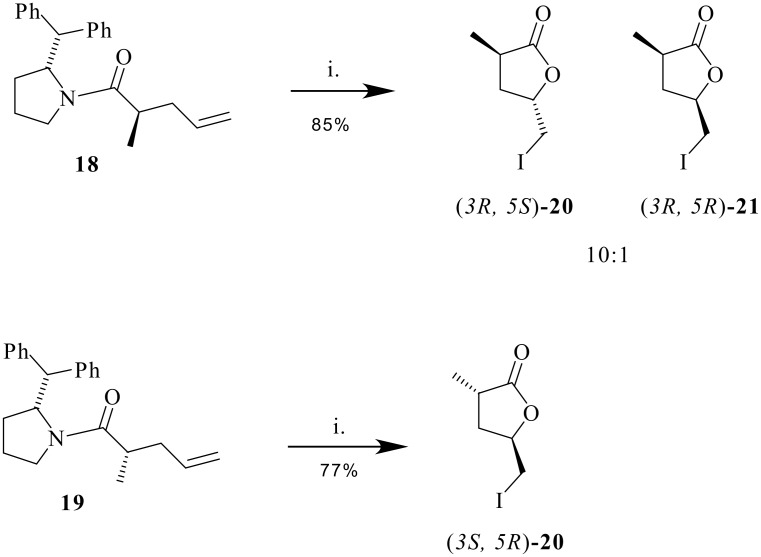
*Reagents*: (a) I_2_, THF/H_2_O, Na_2_S_2_O_3_.

Interestingly iodolactonisation of **17** gave a single product (*3R, 5R*)-**12** ([α]_D_ = +16°) with complete *anti* selectivity and with no indication of the *syn* isomer. A similar reaction sequence was explored for the analogous substrate but without fluorine. Accordingly allyl amine **15** was treated with propionyl chloride to generate a product which was also a mixture of diastereoisomers **18** and **19** in a ratio (3:1) similar to that observed in the fluorinated case. These diastereoisomers were again readily separated by column chromatography to generate homochiral materials. Iodolactonization of **18** furnished the corresponding γ-lactones (*3R, 5S*)-**20** and (*3R, 5S*)-**21**[24] with a significant preference (10:1) for the *anti* diastereoisomer **20** as confirmed by ^1^H-NMR nOe analysis (Scheme 5).

Iodolactonisation of diastereoisomer **19** again generated a single product (*3S, 5R*)-**20**, indicating a much more stereoselective cyclisation.

In order to improve the stereoselectivity of the aza-Claisen rearrangement (*S*)-2(methoxymethyl)pyrrolidine **22** was then explored as the chiral auxiliary.[25] This auxiliary was selected to include a co-ordinating oxygen in place of the bulky diphenylmethane group in **14** to compare steric versus co-ordination effects. Allylation then gave **23** as the required aza-Claisen substrate.

Accordingly allyl pyrrolidine **23** was treated with 2-fluoropropionyl chloride in the presence of Hünig's base and Yb(OTf)_3_. This generated product **24** *as a single stereoisomer*. Reduction of Lewis acid from 1.0 to 0.5 eq did not adversely effect the diastereoselectivity, however a stoichiometry lower than 0.5 eq did compromise the stereoselectivity of the reaction. An analogous reaction with 2-fluorophenylacetyl chloride generated **25**, also as a single stereoisomer. Clearly the co-ordination of the Lewis acid to the ether oxygen is exerting full stereochemical control on the reaction.

**Scheme 6:**
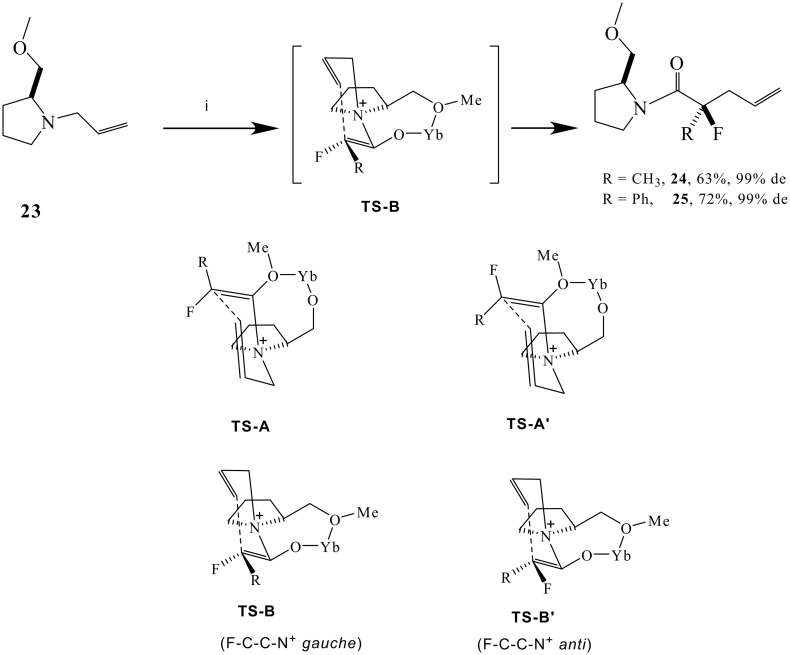
*Reagents*: i *i*Pr_2_EtN, Yb(OTf)_3_, **9** or PhCHFCOCl, DCM, 92%.

This is a highly stereoselective method for the preparation of α-fluoroamides. When the reaction was conducted without a fluorine in the substrate, using propionyl chloride in place of 2-fluoropropionyl chloride, then the diastereoselectivity decreased, generating **26** but in only 75% de. Thus the fluorine as well as the co-ordinating auxiliary appear to play a role in influencing the high diastereoselectivity observed for products **24** and **25**. The reaction presumably progresses *via* a six-membered transition-state as depicted in Scheme 6. There are two possible disastereoisomeric transition states with either the allyl group '*anti*' (**TS-A** and **TS-A'**) or '*syn*' (**TS-B** or **TS-B'**) with respect to the methyl ether substituent of the auxiliary. Models indicate that the **B**-transition states are much more relaxed than the **A**-transition states, with the transient six membered ring perpendicular to the fused five and seven membered rings in **B**. In the **A** transitions states the six and seven membered rings experience considerable steric interactions. It is anticipated also that when the fluorine is *gauche* to the ammonium nitrogen, that this will be significantly stabilising. It has been shown recently that charge dipole interactions [26–27] between vicinal C-F and C-N^+^ bonds significantly stabilise *gauche* over *anti* conformations [28] between these bonds. This effect is large and could clearly influence the diastereoselectivity in a favourable manner with the fluorinated over the non fluorinated substrate. We anticipate that transition **TS-B** derived from the *E* enolate will be lower in energy that **TS-B'** derived from the *Z* enolate, due to a stabilising F-C-C-N^+^ *gauche* relationship in the former, favoured over the *anti* relationship in the latter.

**Scheme 7:**
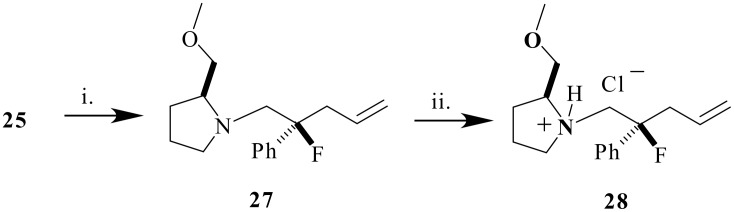
Reagents: i. LiAlH_4_, THF, 99%; ii. HCl-Et_2_O.

**Figure 1:**
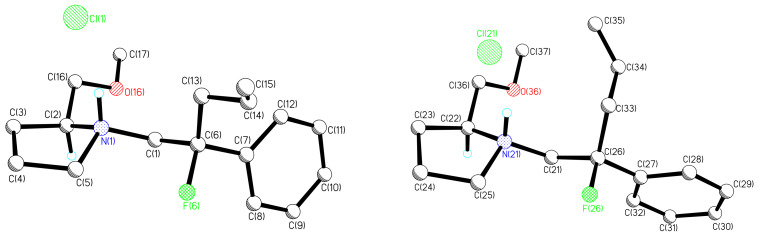
ORTEP drawing of (2*S*, 2'*S*)-**28** showing two crystallographically independent molecules within the unit cell. *Crystal data*: **28** C_17_H_25_NOFCl, *M* = 313.83, monoclinic, space group *P*2_1_, *a* = 7.0446(8), *b* = 23.709(4), *c* = 9.8268(16) Å, *β* = 92.554(4)°, *U* = 1639.6(4) Å^3^, F(000) = 672, *Z* = 4 [two crystallographically independent molecules], *D*_c_ = 1.271 Mg m^-3^, *μ* = 0.242 mm^-1^, 4572 unique data (*R*_merg_ = 0.0194). Conventional *R* = 0.0256 for 4485 reflections with *I* ≥ 2σ, GOF = 1.032. Final *wR2* = 0.0657 for all data (390 refined parameters). The largest differences in the residual maps are 0.191 and -0.201e.Å^-3^. The Flack parameter refined to 0.01(3). Crystallographic data has been deposited with the Cambridge Crystallographic Data Centre as supplementary publication.

In order to assign the absolute stereochemistry of the fluorinated zwitterionic aza-Claisen products, amide **25** was converted to a crystalline derivative for X-ray structure analysis. Treatment of **25** with LiAlH_4_ generated amine **27** which upon HCl-etherate treatment afforded the hydrochloride salt **28** (Scheme 7). The X-ray structure (Figure 1) established the absolute configuration as (2*S*, 2'*S*)-**28** and revealed two crystallographically independent molecules with slightly different conformations in the solid state. Each independent hydrochloride salt displays N-HCl hydrogen bonding [N(1)-H(1n)....Cl(1) 168(2)°, N(21)-H(21n)....Cl(21) 163(2)°].

**Scheme 8:**
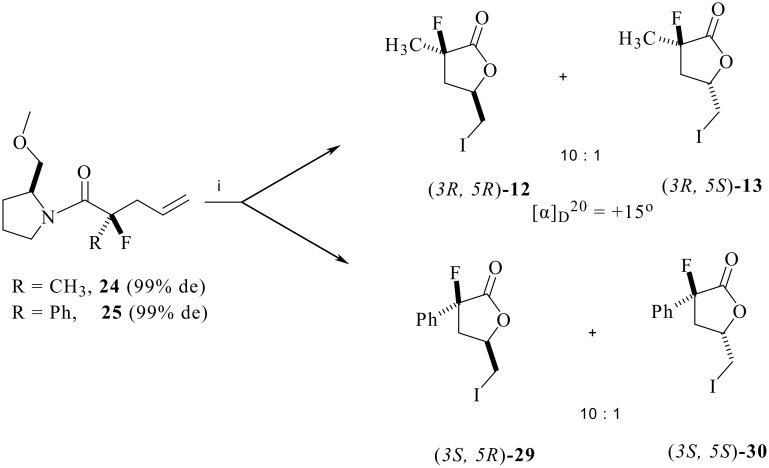
*Reagents*: (a) I_2_, THF/H_2_O, Na_2_S_2_O_3_.

Iodolactonisation of both of the fluorinated products **24** and **25** gave diastereoisomeric γ-butyrolactone products (*3R, 5R*)-**12** and (*3R, 5S*)-**13** and (*3S, 5R*)-**29** and (*3S, 5S*)-**30** respectively, each in a ratio of 10:1 as shown in Scheme 8. The **12/13** mixture had an optical rotation of ([α]_D_ = +15°) indicating a similar absolute stereochemistry to that derived from **17**, thus retrospectively establishing the absolute stereochemistry of **17** and consequently **16**.

## Conclusion

In this study an alternative method for the stereoselective incorporation of α-fluoroamides is demonstrated. The reaction involves a zwitterionic aza-Claisen rearrangement utilising α-fluorocarboxylic acid chlorides with N-allylmorpholine and N-allypyrrolidines. The reaction with N-allylmorpholine is efficient, however by using homochiral pyrrolidine auxiliaries, successful asymmetric reactions were achieved with (*R*)-N-allyl-2-(diphenylmethyl)pyrrolidine **15**, but particularly with (*S*)-N-allyl-2(methoxymethyl)pyrrolidine **23**. Product α-fluoroamides were prepared with very high diastereoselectivities (99%de) and the absolute stereochemistry of these products was determined by derivatisation and X-ray structure analysis. It is notable that with this auxilary the fluorine containing substrates gave higher diastereoselectivities relative to the non-fluorinated counterpart an observation which may have its origin in electronic stabilisation of one diastereoselective transition state as a consequence of the C-F bond. The aza-Claisen products where then subjected to iodolactonisation to generate α-fluoro-γ-butyrolactones, with good diastereoselectivities (~80–100% de). These molecules are useful intermediates for further derivatisation in the area of nucleoside analogue synthesis and the method is complementary to asymmetric electrophilic fluorination strategies for the synthesis of α-fluorocarbonyl compounds.
